# Resolving curling and swelling in cartilage and other deformation-prone tissue explant models: a scalable, simple load-predictive flattening method enabling standardized mechanical compression

**DOI:** 10.3389/fbioe.2026.1753311

**Published:** 2026-07-01

**Authors:** Marlene Lechner, Melanie L. Hart, Alan J. Grodzinsky, Bodo Kurz, Bernd Rolauffs

**Affiliations:** 1 Department of Orthopedics and Trauma Surgery, G.E.R.N. Research Center for Tissue Replacement, Regeneration and Neogenesis, Faculty of Medicine, Medical Center-Albert-Ludwigs-University of Freiburg, Freiburg Im Breisgau, Germany; 2 Center for Biomedical Engineering, Massachusetts Institute of Technology, Cambridge, MA, United States; 3 Department of Anatomy, Christian-Albrechts-University, Kiel, Germany

**Keywords:** articular cartilage, curling, deformation, mechanical injury, post-traumatic osteoarthritis, soft tissue, superficial zone, swelling

## Abstract

**Introduction:**

*Ex vivo* swelling and curling can undermine reproducibility in compression-based injury models.

**Methods:**

We developed a simple, regression-guided flattening step that computes explant-specific loads from each explant’s swelling state to restore native thickness and flatness prior to loading. Using adult articular cartilage, we tested the hypothesis that mitigating initial deformation amplifies injury model readouts and permits standardized injury of the deformation-prone superficial zone (SZ), which many models exclude.

**Results:**

Discs spanning 250–1200 µm exhibited curling; 250 µm SZ discs were chosen for calibration. Post-equilibration thickness (T_2_) strongly predicted the load required to restore native thickness (LT_0_), and nonlinear regression yielded a practical load-prediction equation, refined with an expanded dataset (n = 129 discs). Applying the predicted flattening load immediately before standardized compression (50%–65% strain, 100%·s^-1^) did not affect chondrocyte viability on its own. When combined with injury, it amplified canonical readouts - lower viability and higher apoptosis - with effects concentrated in the upper superficial zone, notably within the top 44  μm at 24 h and with apoptosis persisting to 96 h. Click-chemistry EdU labeling revealed injury-induced proliferation: SZ-wide S-phase entry, most pronounced at 96 h in adult, mechanically mature cartilage without exogenous growth factors.

**Discussion:**

Together, regression-guided flattening restores native geometry, standardizes the mechanical stimulus, increases readout reliability, and enables inclusion of the clinically important SZ. Because deformation artifacts are common across hydrated soft tissues, and prior pre-compression practices were uncalibrated, this framework establishes a cartilage-based proof of concept for regression-guided flattening that may be extendable to other deformation-prone tissues through tissue-specific recalibration and experimental validation, thereby providing a more standardized and generalizable route to faithful *ex vivo* injury studies.

## 1 Introduction

Mechanical tissue injury models simulate mechanical stresses and are essential not only for understanding strain-related damage but also mechanisms of post-injurious degeneration and inflammation and the effectiveness of treatment strategies. Despite the prevalence of such models, few address the physical deformations that tissues undergo *ex vivo* when immersed in aqueous media, such as curling or swelling, although these artifacts can compromise mechanical loading and the accuracy and reproducibility of mechanical injury studies. Such artifacts are reported in articular cartilage (Setton et al., 1998; Maroudas, 1976; Brown et al., 2020; Brown et al., 2022; Wan et al., 2010), meniscus (Andrews et al., 2015), tendons (Screen et al., 2006; Safa et al., 2017), brain (Hrabetová et al., 2002; Nosrati et al., 2025; Lang et al., 2014), cornea (Dias and Ziebarth, 2015), and vascular walls (Chuong and Fung, 1986), while skin (Waal, 2021) deformation is associated with shrinkage. These artifacts might alter explant geometry and boundary conditions, yet most mechanical injury models do not correct for them. Subsequently, a method to mitigate *ex vivo* tissue deformation prior to mechanical loading would be widely applicable and improve model reliability. Here, we developed a practical nonlinear-regression–guided approach which calculates the load required to flatten swollen, curled tissue back to its original tissue thickness, and subsequently applies controlled flattening. This simple approach resolves *ex vivo* deformation, while enabling uniform mechanical loading. We hypothesized that this would in turn significantly improve model reliability, which we subsequently tested using a standardized injurious compression model of articular cartilage.

Articular cartilage was chosen as model tissue since it is susceptible to traumatic injury, which accelerates the development of post-traumatic osteoarthritis (PTOA), a common degenerative joint disease (Brown et al., 2006). PTOA develops much earlier than typically seen in age-related osteoarthritis (OA) (Brown et al., 2006; Muthuri et al., 2011; Lohmander et al., 2007; Khella et al., 2021; Roos et al., 1995), progresses through mechanical overload, chronic, persistent, low-grade inflammation (Khella et al., 2021), and often initiates in the superficial zone. The superficial zone is also the most clinically relevant region: it is the primary site of early PTOA initiation, bears load, regulates shear, and is highly susceptible to compressive damage (Bauer and Jackson, 1988; Ewers et al., 2001; Rolauffs et al., 2010a; Chen et al., 2003; Jones et al., 2009; Bartell et al., 2015). It contains the most cell-dense zone with specialized cell arrangements indicating early degeneration (Tscha et al., 2021; Tschaikowsky et al., 2021) and is important for advancing our understanding to investigate or modulate post-injurious events. Many ex vivo cartilage mechanical injury models exclude the superficial zone to reduce the swelling potential of the ECM and create a level explant surface (Rolauffs et al., 2010a; Imgenberg et al., 2013; Kurz et al., 2023; Patwari et al., 2003; Patwari et al., 2004; Sui et al., 2009) or replicate OA conditions by removing the superficial zone or investigate OA cartilage (Brown et al., 2022). In contrast, we retain the superficial zone in the here introduced nonlinear-regression–guided approach for correcting ex vivo deformation.

Retaining the superficial zone remains technically challenging in many cartilage injury models because it is the region most prone to swelling-induced curvature, making it difficult to achieve uniform loading. As a result, many *ex vivo* models remove the superficial zone to obtain a flat explant surface, which would omit superficial zone-specific responses. To address this gap, we developed a nonlinear-regression–guided approach for reversing swelling-induced deformation prior to loading, enabling precise, reproducible mechanical stimulation while preserving the superficial zone and avoiding swelling-induced surface contact alterations and spatially heterogeneous injurious strain fields. This framework allows us to test whether correcting *ex vivo* deformation improves model fidelity and enables the inclusion of deformation-prone superficial tissues that are typically excluded from standard injury paradigms.

Our findings demonstrate that mitigating *ex vivo* deformation, combined with standardized mechanical injury, generates a physiologically relevant and reproducible model for tissue explants prone to swelling and curling. In cartilage, it preserves the clinically important superficial zone while enhancing injury effects and biological relevance. Beyond facilitating *ex vivo* PTOA research, this work establishes a cartilage-based framework that may be extendable to other swelling- and deformation-prone soft tissue explants through tissue-specific recalibration and experimental validation.

## 2 Materials and methods

### 2.1 Production of articular cartilage discs

Full thickness cartilage explants from the load-bearing areas of articular cartilage were excised from the femoro-patellar grooves of adult 2–3 year old bovines, which were obtained from the Emil Färber GmbH slaughterhouse (Freiburg, Germany) within 12 h after slaughter. Samples were harvested from completely closed knee joints under sterile conditions (Microbiological Safety Workbench, ENVAIR eco safe Comfort, ENVAIR, Emmendingen, Germany). A sized 21 scalpel (PFM Medical GmbH, Köln, Germany) was used to cut cartilage strips parallel to the cartilage surface, cutting directly above the bone. Cartilage discs with varying thicknesses including the intact superficial zone were obtained as follows. Using a 4 mm dermal biopsy punch (PFM Medical GmbH, Köln, Germany), cartilage cylinders were produced and cut into discs (Rolauffs et al., 2010a). These were measured using a sterile caliper (Henry Schein Dental Deutschland GmbH, Langen, Deutschland) and further processed to generate discs with 250, 300, 400, 500, 600, 700, 800, 900, 1,000, 1,100, and 1,200 µm thickness, using a custom-made cutting block with various insets, e.g., 300 and 600 µm depths. Specifically, the cartilage cylinders were placed in the cutting block in the inset with desired depth, surface down, held with a small spatula and discs were cut using a sterile razor blade (Wilkinson Sword GmbH, Solingen, Germany). Explants were sliced perpendicular to the longitudinal axis to produce disc-shaped explants. Immediately after cutting, the discs were transferred to a 96-well F-Bottom tissue cell culture plate (Greiner Bio-One International GmbH, Kremsmünster, Austria) containing 200 µL explant medium in DMEM with 10% FBS, 10 nM HEPES, 1 nM sodium pyruvate, 0.1 mM non-essential amino acids, 0.4 mM proline, 20 μg/mL ascorbic acid plus antibiotics per well with the surface facing up and incubated at 37 °C and 5% CO2 (CO_2_-Incubator, Galaxy 170 R, Eppendorf SE, Hamburg, Germany). The degree of curling of cartilage discs with varying thicknesses was defined as described in the results section.

### 2.2 Curling degree assignment, thickness measurements, and injurious compression

**FIGURE 1:**
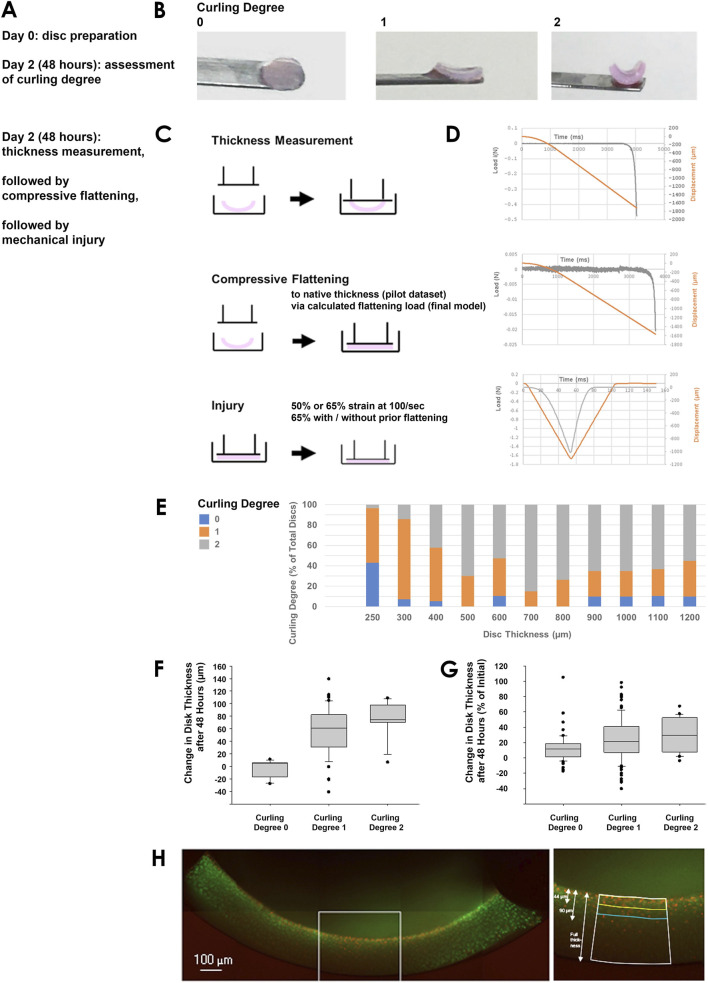
*Ex vivo* deformation of tissue explants: curling and swelling **(A)** Small “workflow” schematic **(B)** Representative photographs of cartilage discs with the curling degrees of 0, 1, and 2 **(C)** Schematic overview on thickness measurements, compressive disc flattening, and injury application. The pink shapes represent discs in their curled, flattened and injured state. The position of the top part of the loading device relative to the curled disc indicates how the thickness of curled discs was measured **(D)** Representative displacement and load curves recorded during thickness measurements, compressive disc flattening, and injury application (strain rate: 100%/s, final strain: 50%) **(E)** Distribution of curling degrees (in % of all discs). The following number of discs was assessed per thickness category: 250 µm: n = 28, 300 µm: n = 28; 400 µm: n = 19, 500 µm: n = 20, 600 µm: n = 27, 700 µm: n = 20, 800 µm: n = 19, 900 µm: n = 20, 1,000 µm: n = 20, 1,100 µm: n = 19, and 1,200 µm: n = 20 **(F,G)** Box plots indicating change in thickness (ΔT = T_2_ -T_0_) in µm and in percent of initial thickness (right) as a function of curling degree. The box plots present the median as line and the 25th and 75th percentiles, the whiskers give the 10th and 90th percentiles, and the round symbols, if present, indicate outliers. Curling degree 0: n = 48, curling degree 1: n = 104, curling degree 2: n = 17) **(H)** Left: representative image of half of a superficial zone disc in a side view indicating one of three tiles that were selected per superficial zone disc. Each tile covered the entire superficial zone disc thickness. Right: the selected tile was subdivided into three polygonal regions of interest (ROI), using one ROI for the entire 250 µm superficial zone discs (white), one for the uppermost 90 µm of the superficial zone (blue) and another for the uppermost 44 µm (yellow). Scale bar: 100 µm.

The degree of curling was assigned using the following definitions: 0 indicated a flat disc, one indicated a slightly curled disc, and two indicated a heavily curled disc (timeline and representative photographs are shown in Figures 1A,B). For measuring disc thickness and applying mechanical loads (Figures 1C,D), an incubator-housed, atmosphere- and temperature-controlled loading device (IncuDyn, M.I.T. Biomedical Engineering Center, Boston, United States) (Frank et al., 2000) capable of applying axial deformations and sinusoidal rotations to cultured tissue explants was used at 37 °C and 5% CO2. Before usage, the device was calibrated for displacement and load using an empty loading chamber and a ramp compression (100 μm/s velocity, automated compression interrupt at −0.02 N offset load). Each disc was placed in the center of the well of the loading chamber under sterile conditions and with 20 µL of explant medium to prevent drying-out. To flatten discs back to their original state by compression, the thickness of each disc was measured on day 0 immediately after preparation (T0) and on day 2 (T2; 48 h after equilibration), as described above. Using the measured change in thickness (T_2_–T_0_), each disc of the pilot disc set was compressed back to its original thickness (T_0_) at 100 μm/s following a 2-s acceleration phase, while displacement and load were continuously recorded. In the extended disc set used for generating the final regression model, the calculated flattening load was applied for flattening discs. After loading device re-calibration and successful flattening of each superficial zone disc back to its original thickness to mitigate day 2 *ex vivo* deformation, a single injurious compression (Figures 1C,D “Injury” Panel) was applied with a strain rate of 100%/s and a final strain of either 50% or 65%, depending on the experimental group. Post-injury, samples were transferred to fresh explant medium with the superficial zone surface facing up and assessed as described below.

### 2.3 Cell viability in regions of interest within superficial zone discs

For live-dead imaging 24 and 96 h after injury, a staining solution was prepared using explant medium containing Calcein (Thermo Fisher Scientific, Waltham, United States) to stain the viable and Propidium Iodide (Sigma-Aldrich Chemie GmbH, Taufkirchen, Germany) to stain the dead cells. Each disc was transferred to a 96-F-bottom well plate, containing 100 µL of staining solution (1:10 ratio dilution for Calcein and 1:500 ratio dilution for PI) per well and incubated in a 37 °C and 5% CO2 incubator for 1 h in darkness. After the incubation period, the discs were washed twice with fresh medium and individually transferred to a sterile Petri dish and cut in two-halves of the same size, perpendicular to the articular surface (and, in curled specimens, perpendicular to the direction of curvature). A small spatula was used to place both halves on the bottom of a fresh 96-well plate with the cut surface flat on the bottom. 20 μL of fresh explant medium was added to prevent drying out. The superficial zone discs were imaged using an Axio Observer. Z1 inverse microscope (Carl Zeiss AG, Oberkochen, Germany) and the ZEN blue software. Imaging and region selection were performed manually. After cutting each superficial zone disc in half, three tiles were manually selected for each half-disc based on optimal focus and inclusion of the full tissue thickness. Within each tile, three polygonal regions of interest (ROIs) were manually drawn: (i) the full 250 µm disc thickness, (ii) the top 90 μm, and (iii) the top 44 µm. The 44 µm region was selected to represent the immediately surface-adjacent portion of the superficial zone. ROIs excluded outer edges and out-of-focus regions. For each ROI, viable and dead cells were quantified as described above. For each depth zone (full thickness, top 90 μm, top 44 µm), the corresponding ROI from each tile was treated as one observation, yielding up to three technical replicates (tiles) per half-disc and depth zone. These ROI-level values were used directly for plotting and statistical analyses, as analyzing tile-level ROIs rather than pre-averaged disc means allows us to capture local spatial variability within the superficial zone. Thus, ROI-level values were treated as independent technical observations. Images were processed in ImageJ by enhancing contrast within each ROI and applying a Gaussian blur filter (σ = 2.0) for de-noising. Viable and dead cells were counted using the “Find Maxima” function, with prominence adjusted manually in preview mode to reduce artifacts. Cell counts were normalized per unit area, and viability was defined as (viable cells per µm^2^)/(viable cells per µm^2^ + dead cells per µm^2^). In all figures, “n” denotes the number of tiles (technical replicates). Each half-disc contributed up to three tiles, and each tile yielded three ROIs (one per depth zone).

### 2.4 Cell death and apoptosis of chondrocytes

The amount of apoptotic cells and dead cells was visualized 24 and 96 h after injury using Annexin V conjugate (Thermo Fisher Scientific, Waltham, United States)/Propidium Iodide (PI; Sigma-Aldrich Chemie GmbH, Taufkirchen, Germany) co-staining following the manufacturer’s protocol with the following slight adaptations for cartilage explants, based on preliminary experiments (data not shown). 25 μL of the Annexin V conjugate and 0.1 µL PI were added to a 100 µL of 1X Annexin-binding buffer and incubated for 15 min. Cartilage explants were washed in Annexin-binding buffer twice and added to a 96-well-F-bottom plate containing 62.5 µL of the staining solution per well and incubated under the exclusion of light for 1 h. Cartilage explants were washed with 100 ul of Annexin-binding buffer twice and placed in a fresh well plate containing 100ul of buffer per well. Superficial zone discs were then cut in half and imaged and analyzed using the same tile- and ROI-based workflow described in the section “Cell viability in regions of interest within superficial zone discs.” Thus, up to three tiles per half-disc and three ROIs per tile (one per depth zone) were obtained, and ROI-level values were used as technical replicates for plotting and statistical analyses. Tiles were selected according to predefined criteria to ensure consistent sampling of the superficial zone. Regions affected by edge artifacts, incomplete tissue representation, strong local curvature, or imaging distortions were excluded from analysis. Apoptotic cells (Annexin V), dead cells (PI), and the ratio of apoptotic cells to total cells were quantified per µm^2^. Cells positive for Annexin V were considered early apoptotic, whereas PI-positive cells were considered dead.

### 2.5 Proliferation of chondrocytes

The number of proliferating cells was measured 24, 48, 72, and 96 h after injury using Click-iT™ EdU (Thermo Fisher Scientific, Waltham, United States). Although the manufacturer’s protocol is optimized for cell lines, and previous studies have used EdU on embryonal tissue (Click-iT™ EdU, 2023), protocol adaptations were required for cartilage explants. Based on preliminary experiments (data not shown), explant medium containing 50 µM EdU was used to label proliferating cells. After injurious compression, cartilage discs were transferred to a 96-well plate containing 200 µL of EdU medium and incubated at 37 °C and 5% CO_2_. Samples remained in this EdU-containing medium until fixation at their respective time points; for discs assigned to later time points (48, 72, 96 h), the EdU medium was refreshed on day 2. At each time point, superficial zone discs were fixed in 4% paraformaldehyde for 2 h, washed twice in 3% bovine serum albumin (BSA) in DPBS, permeabilized in 1× Triton™ X-100 for 4 h, washed again in 3% BSA, and stored overnight in DPBS. Click-iT™ EdU reaction buffer (100 µL per well) was applied for 1 h at room temperature in the dark. Discs were washed twice in 3% BSA and counterstained with Hoechst for 1 h. After two additional washes in 3% BSA, discs were placed in fresh DPBS, cut in half, and imaged as described above, with 20 µL DPBS added per well to prevent drying. Hoechst staining confirmed complete tissue fixation and permeabilization (data not shown). Disc contours were traced using tissue autofluorescence to compute disc area (µm^2^), and EdU-positive cells were counted manually and normalized per unit area. Due to the low number of proliferating cells, counts were performed manually. For the proliferation analysis, each half-disc constituted one biological replicate; thus, n denotes the number of half-discs, and tile-level technical replicates were not evaluated separately for this assay.

### 2.6 Statistical analyses

Data handling was performed in Microsoft Excel 2021, and statistical analyses and figure generation were conducted in SigmaPlot Version 14.0 (Systat, Chicago, United States). Summary statistics are reported as percentages or mean ± SEM where appropriate. Box plots display the median (center line), 25th and 75th percentiles (box), 10th and 90th percentiles (whiskers), and outliers (dots). Pearson correlation tests were used to assess relationships between initial and post-swelling disc thicknesses and between the change in thickness (T_2_–T_0_) and the load required for flattening. Pearson correlation was used because all variables were continuous and the relationships appeared monotonic and approximately linear in scatterplots, which supports the use of Pearson’s r even when the best-fitting regression model (below) was nonlinear. Regression analyses (linear, exponential, logarithmic, and polynomial) were performed using SigmaPlot and TableCurve 2D (Version 5.1, Systat, Chicago, United States), and R^2^ values were reported as indicators of fit quality (Rolauffs et al., 2013). For group comparisons, linear mixed-effects models were used to account for the nested experimental structure and cow-associated biological variability. The experimental group was modeled as a fixed effect, and cow was included as a random intercept. Pairwise group comparisons were performed using estimated marginal means with multiplicity correction. This approach allowed ROI- and tile-level measurements to be retained for visualization and statistical modeling while avoiding the assumption that all ROIs represented independent biological replicates (pseudoreplication). Mixed-effects analyses were performed in R using lme4, lmerTest, and emmeans. Curling degree distributions were analyzed using cumulative link mixed-effects models (ordinal mixed-effects models) implemented in the ordinal package in R. Curling degree was modeled as an ordered categorical outcome, disc thickness as fixed effect, and donor as random intercept. Overall model significance was assessed using likelihood-ratio tests, and pairwise comparisons between thickness groups were performed using estimated marginal means with Holm correction for multiple testing. Correlations and group differences were considered statistically significant for p < 0.05.

## 3 Results

### 3.1 Curling and swelling of cartilage discs

Figure 1A gives a small “workflow” schematic, whereas Figures 1B–D illustrate relevant workflow points. We first determined if disc thickness had an effect on the curling behavior 48 h after equilibration. Across thicknesses, all groups exhibited some curling (Figure 1E). Discs cut to 250 µm thickness showed the highest proportion with curling degree 0 and the fewest with degree two and, thus, showed the lowest tendency to curl. Since this thickness corresponds to superficial-zone–only explants, we refer to this group as ‘superficial zone discs,’ which we used for subsequent analyses. To account for donor-associated variability, curling degree was analyzed using an ordinal mixed-effects model with donor included as a random intercept. Mixed-effects modeling indicated moderate donor-associated variability in curling behavior (ICC ≈0.20). The overall effect of thickness on curling degree distribution was highly significant (likelihood-ratio test, p < 0.0001). Pairwise comparisons with multiplicity correction demonstrated that 250 µm discs differed significantly from discs of 400–1,200 µm thickness (all adjusted p < 0.01), whereas the difference between 250 and 300 µm discs was not significant after donor adjustment. Thus, discs with a thickness of 250 µm showed the lowest tendency to curl. Subsequent experiments, focusing on this group, showed that the superficial zone discs - initially cut to 250 µm - changed in thickness from 190–504 µm 48 h after disc generation (Figure 1E). Linear mixed-effects models accounting for donor-associated variability (ICC ≈0.14) revealed a significant association between curling degree and thickness increase (likelihood-ratio test, p < 0.001). Pairwise comparisons showed that discs with curling degree two exhibited significantly greater absolute (Figure 1F) and relative (Figure 1G) thickness increases compared to discs with curling degrees 0 and 1 (adjusted p < 0.01), whereas no significant difference was observed between curling degrees 0 and 1. Interestingly, donor-associated variability contributed more strongly to curling behavior (ICC ≈0.20) than to swelling magnitude itself (ICC ≈0.14).

### 3.2 Swelling, correlation and regression analysis for calculating flattening loads to mechanically mitigate *ex vivo* deformation prior to standardized injurious compression

Visual inspection confirmed that mechanically flattening the discs yielded flat discs as expected. However, when returned to explant medium, discs re-curled within seconds to their original state without any macroscopically visible damage. This motivated the development of a procedure to mechanically flatten discs immediately prior to injurious compression and to calculate explant-specific flattening loads using a regression-based prediction model (Table 1). To establish this model, a preliminary subset of discs with 250 µm thickness with a curling degree of 0 after cutting was analyzed (n = 86 discs, 8 animals). Of these, 20 discs (23.2%) decreased in thickness over 48 h, 5 discs (5.8%, only occurring in discs with curling degrees 0 and 1) exhibited minor slipping during measurement before the completion of load application. The remaining 61 discs (71%) increased in thickness over time and these were included in the subsequent steps. Individual disc thicknesses were measured on day 0 immediately after preparation (T_0_) and again on day 2 after equilibration (T_2_). The change in thickness (T_2_–T_0_) was calculated, and the load required to compress each disc back to its original thickness on day 2 (L_t0_) was recorded using a linear compression at 100 μm/s. The loading device reports force in gram-force equivalents and uses a small threshold force of −2 g (≈−0.0196 N) as its calibrated contact-detection point; the negative sign denotes the compression direction. Disc thickness was therefore defined as the displacement at which this threshold was reached.

**TABLE 1:** Parameters measured for calculating loads for disc flattening.

Parameter	Explanation	Equation	Required variables
Cal T_0_	Calculated native (day 0) thickness	Cal T_0_ = 0.5737 x T_2_ + 71.717	Measured T_2_
Cal(T_2_ -T_0_)	Calculated change in thickness/swelling magnitude	Cal(T_2_-T_0)_ = T_2_ – CalT_0_	Measured T_0_
Cal L_0_ (from regression)	Calculated flattening load	Cal L_0_ = −0.3708 x Cal(T_2_-T_0_) −3.2564	Cal(T_2_-T_0)_
Cal L_0_ (final merged equation)	Pilot equation for calculating flattening load/flattening load expressed directly as a function of T_2_	Cal L_0_ = −0.3708 x [T_2_ – (0.5737 x T_2_ + 71.717)] – 3.2564	Measured T_2_

Abbreviations: Cal, calculated, T, thickness, L, load, 0 = day 0, 2 = day 2.

T_0_ and T_2_ were directly measured. Using their regression relationships, the intermediate values Cal T_0_ and Cal(T_2_–T_0_) were computed. The final merged equation (bottom row) provides a single-input predictor for Cal L_0_ that requires only the post-equilibration thickness T_2_.

Pearson correlation tests revealed significant relationships between disc geometry and flattening load. T_0_ and T_2_ showed a correlation coefficient of 0.811 (p < 0.001), and the swelling magnitude (T_2_–T_0_) and L_t0_ showed a coefficient of 0.651 (p < 0.001). These results supported using post-equilibration thickness (T_2_) as the predictor for the flattening load.

To generate an initial regression model on a pilot dataset, discs were treated as independent observations because the goal was to model the population-level thickness–load relationship, and swelling/curling behavior varied disc-specifically rather than animal-specifically. The regression equations used to estimate T_0_ from T_2_, calculate swelling, and predict the flattening load L_t0_ are summarized in Table 1. Briefly, a linear model was first used to estimate T_0_ from T_2_, and the predicted swelling (T_2_–T_0_) was then regressed against the measured flattening load. This yielded a single-input predictor for L_t0_ that depends only on T_2_ and was later refined using an expanded dataset.

### 3.3 Flattening superficial zone discs in conjunction with injurious compression reduced viable cell numbers and increased cell death in the most superficial layers

To test the general utility of the load-prediction equation (Table 1), we applied the calculated flattening load to each disc prior to a single injurious compression (final strains of 65% or 50% of the measured thickness; strain rate 100%/s) and assessed whether flattening influenced post-injury cell viability within selected tiles and ROI (Figure 1H). To prevent re-curling of the discs after flattening, the platen was not elevated between the flattening and injury steps. Injurious compression was applied immediately following flattening while the discs remained mechanically constrained, thereby minimizing variability caused by rapid tissue re-curling. Viable and dead cells were quantified within three depth-defined regions of the same discs: the full 250 µm disc thickness, and the uppermost 90 μm and 44 µm regions.

**FIGURE 2:**
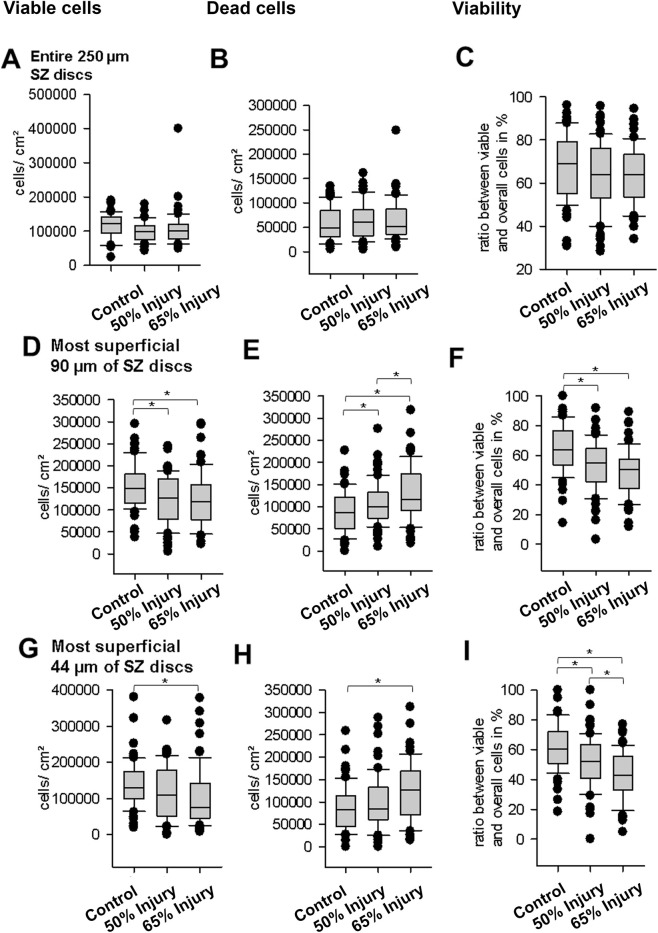
Viable and dead cells after flattening of superficial zone discs followed by single injurious compression 24 h after injury **(A,D,G)** Results for viable cells that were stained with Calcein **(B,E,H)** Results for dead cells that were stained with PI **(C,F,I)** Results for viability multiplied by 100. Control: n = 69 from 5 donors, 50% Injury: n = 72 from 5 donors, 65% Injury: n = 66 from 5 donors. Here, n denotes the number of analyzed tiles (and therefore the number of analyzed ROIs per depth zone: 250 μm, 90 μm, and 44 µm). Because each half-disc yields three tiles, n/3 corresponds to the number of analyzed half-discs. The * indicates a significant difference between groups (p < 0.05).

24 h after injury (Figure 2), viable and dead cell counts and viability within the full 250 µm superficial-zone discs did not differ significantly between groups (Figures 2A–C). In the upper 90 µm (Figures 2D–F), both injury groups showed significantly reduced viable cell numbers and viability compared with controls, while dead cell numbers were significantly increased in the 50% and 65% injury groups. In the uppermost 44 µm layer (Figures 2G–I), viability was significantly reduced in both injury groups, accompanied by significantly increased dead cell numbers in the 65% injury group. In addition, viable cell numbers were significantly reduced in the 65% injury group compared with controls.

**FIGURE 3:**
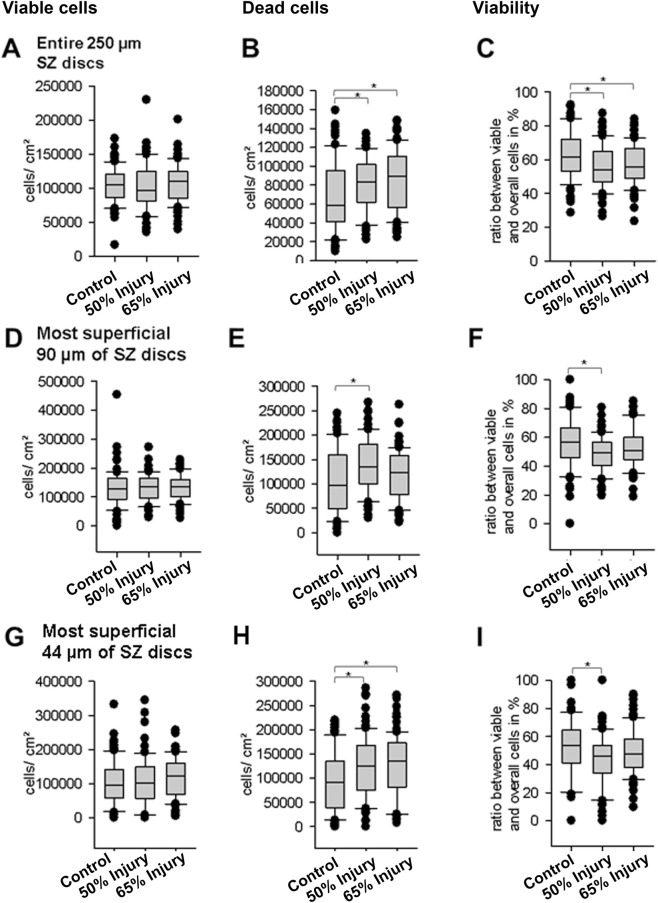
Viable and dead cells after flattening of superficial zone discs followed by single injurious compression 96 h after injury **(A,D,G)** Results for viable cells that were stained with Calcein **(B,E,H)** Results for dead cells that were stained with PI **(C,F,I)** Results for viability multiplied by 100. Control: n = 87 from 5 donors, 50% Injury: n = 81 from 5 donors, 65% Injury: n = 90 from 5 donors. Here, n denotes the number of analyzed tiles (and therefore the number of analyzed ROIs per depth zone: 250 μm, 90 μm, and 44 µm). Because each half-disc yields three tiles, n/3 corresponds to the number of analyzed half-discs. The * indicates a significant difference between groups (p < 0.05).

96 h after injury (Figure 3), the pattern differed from the 24-h response. Across the full 250 µm of the discs (Figures 3A–C), viable cell numbers no longer differed significantly between groups, whereas dead cell counts were significantly elevated and viability was significantly reduced in both injury groups. Similar effects were observed within the upper 90 µm region (Figures 3D–F). In the uppermost 44 µm region (Figures 3G–I), viability in the 65% group did not differ significantly from controls, although dead cell counts remained significantly elevated. Overall, injury-related changes were most pronounced at 24 h and within the most superficial regions of the discs. By 96 h, elevated cell death persisted, consistent with prior observations in cartilage injury models (Quinn et al., 2001; Delco et al., 2018; Morel and Quinn, 2004).

### 3.4 Refinement of a regression-driven equation for calculating flattening loads to mechanically mitigate *ex vivo* deformation prior to standardized injurious compression

After establishing in a pilot model (above) that disc flattening prior to injurious compression was feasible and enhanced the detectability of injury-related effects on viability and cell death, we refined the regression model using a substantially larger disc subset. For the below described refinement process, the sample size was increased to 129 discs cut to an initial 250 µm thickness (n = 14 animals). All regressions were performed using TableCurve 2D.

#### 3.4.1 Step 1: predicting native thickness (T_0_) from post-equilibration thickness (T_2_).

A nonlinear model best described the relationship between the post-equilibration thickness and the estimated native thickness:

T0=‐75.9646+3.1622×T2^0.5×lnT2

This model showed good explanatory performance (R^2^ = 0.769, fit standard error = 33.1) and provided a practical mapping from T_2_ to T_0_, indicating that thicker post-swelling discs tended to have proportionally greater native thickness.

#### 3.4.2 Step 2: predicting the flattening load (LT_0_) from swelling (T_2_ – T_0_).

A linear model was fitted between the required flattening load and the measured swelling:

$${\text{LT}}_{0}=1.5902\;‐\;0.5923\times \left(T_{2}‐T_{0}\right)$$

Although the correlation was weaker than in step 1 (R^2^ = 0.349, fit standard error = 33.92), this model still provided a usable mapping from swelling to flattening load.

#### 3.4.3 Step 3: constructing a single-input operational predictor requiring only T_2_.

To obtain a practical predictor requiring only one measurement, the predicted T_0_ from Step 1 was substituted into the flattening-load model from Step 2. The resulting operational predictor was:

LT0=1.59‐0.59×T2‐  ‐75.96+3.16×T2^0.5×lnT2

Regression coefficients are rounded to four digits for readability. Here, LT_0_ represents the load required to flatten a disc back to its native thickness prior to injury, and T_2_ is the disc thickness measured 48 h post-dissection. This final predictor was subsequently used for all experiments.

### 3.5 Application of pre-injurious superficial zone disc flattening increased injurious compression effects

In the next study phase, we applied the refined equation to test whether the flattening step enhanced the biological effects of injurious compression. Because viable cell numbers at 96 h post-injury did not differ significantly from controls in the initial experiment, we also assessed whether this could be explained by injury-induced proliferation.

#### 3.5.1 Effect on cell numbers and viability

**FIGURE 4:**
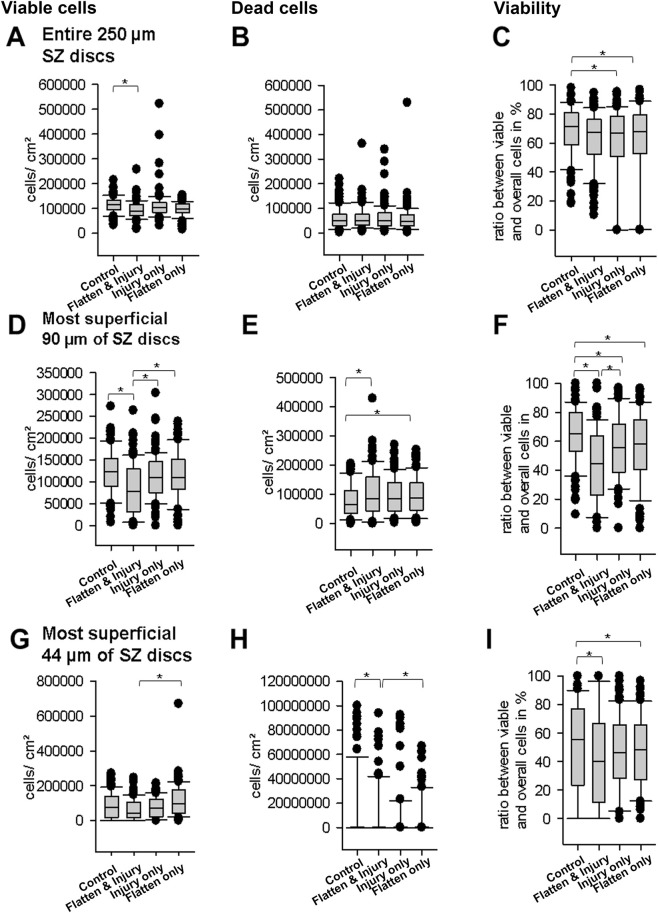
Viable and dead cells after flattening of superficial zone discs, single injurious compression (65%) or the combination 24 h after injury **(A,D,G)** Results for viable cells that were stained with Calcein **(B,E,H)** Results for dead cells that were stained with PI **(C,F,I)** Results for viability multiplied by 100. Control: n = 132 from 8 donors, flattening and injury: n = 111 from 8 donors, injury only: n = 135 from 8 donors, flattening only: n = 123 from 8 donors. Here, n denotes the number of analyzed tiles (and therefore the number of analyzed ROIs per depth zone: 250 μm, 90 μm, and 44 µm). Because each half-disc yields three tiles, n/3 corresponds to the number of analyzed half-discs. The * indicates a significant difference between groups (p < 0.05). Injury included a single traumatic compression (65% final strain, 100%/s strain rate).

To determine if the flattening step increased the effects of injurious compression, the groups control (no injury and no flattening), injury after flattening, injury without flattening and flattening only were compared (Figure 4). Across the full 250 µm disc thickness (Figures 4A–C), the flattened and injured group showed significantly lower viable cells vs. the control group while none of the groups showed a significant difference in dead cells. In contrast, viability was significantly reduced in the injury-only and flattening-only groups, compared with controls. In the uppermost 90 µm disc region (Figures 4D–F), the flattened and injured group showed significantly reduced viable cell numbers and significantly reduced viability, compared to the control, flattening-only and injury-only groups. In this region, dead cell numbers were significantly increased in the flattening and injury group compared with controls. In the uppermost 44 µm disc region (Figures 4G–I), the flattened and injured group exhibited significantly lower viable cells vs. the flattening-only group. Dead cell numbers were significantly different between the flattening and injury group compared with both the control and flattening-only group. Viability was significantly reduced in the flattening and injury group and in the flattening-only group, compared with the control group. Overall, flattening alone did not substantially impair viability, whereas flattening combined with injury consistently enhanced injury-associated effects, particularly within the most superficial regions of the discs. Altogether, this was in accordance with prior studies on osteochondral plugs showing that injury-associated reductions in viability are most pronounced within the superficial zone (Rosenzweig et al., 2012; Quinn et al., 2001; Clements et al., 2001). Collectively, these results indicate that flattening of curled and swollen discs prior to injury enhanced the detectable cellular consequences of injurious compression.

#### 3.5.2 Effect on cell death and apoptosis

**FIGURE 5:**
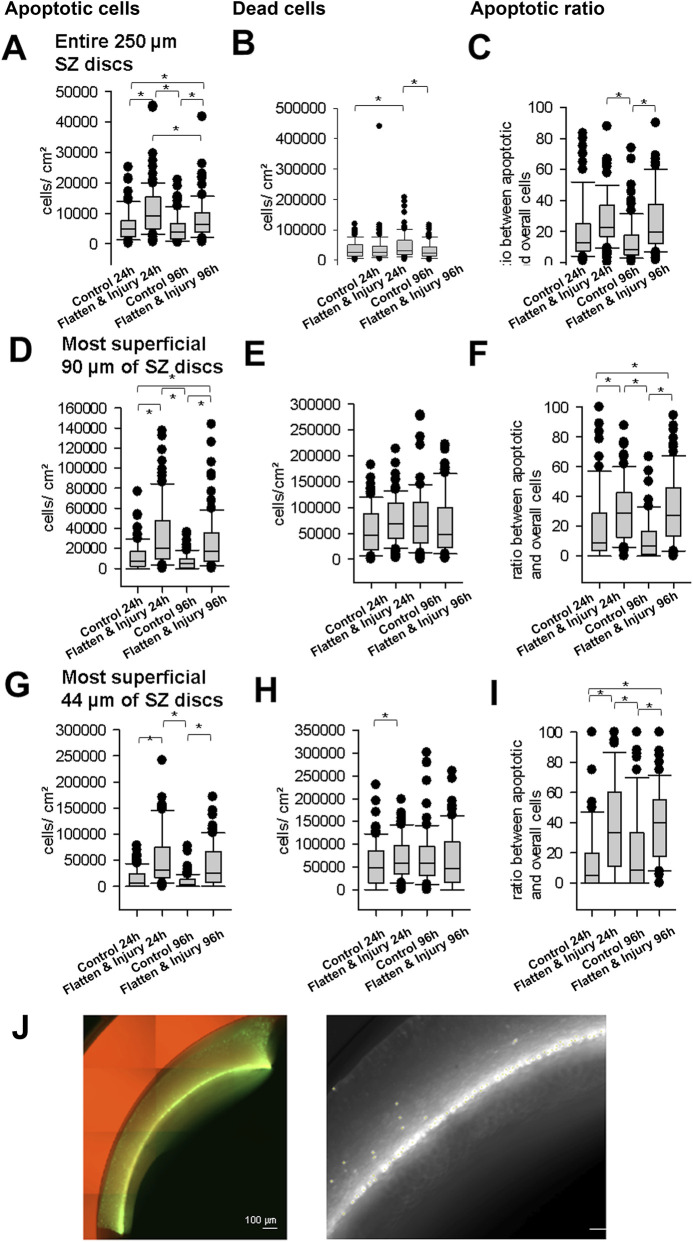
Apoptotic and dead cells after flattening of superficial zone discs followed by a single injurious compression 24 h and 96 h after injury (65%) **(A,D,G)** Results for apoptotic cells that were stained with Annexin V **(B,E,H)** Results for dead cells that were stained with PI **(C,F,I)** Results for the calculated ratio (apoptotic cells/(apoptotic cells + all cells)) multiplied by 100. Control 24 h: n = 87 from 8 donors, flattening and injury 24 h: n = 93 from 8 donors, control 96 h: n = 72 from 8 donors, flattening and injury 96 h: n = 87 from 8 donors. Injury included a single traumatic compression (65% final strain, 100%/s strain rate). Here, n denotes the number of analyzed tiles (and therefore the number of analyzed ROIs per depth zone: 250 μm, 90 μm, and 44 µm). Because each half-disc yields three tiles, n/3 corresponds to the number of analyzed half-discs. The * indicates a significant difference between groups (p < 0.05). Injury included a single traumatic compression (65% final strain, 100%/s strain rate) **(J)** Representative images of superficial zone discs subjected to flattening and injury after 96 h stained with Annexin V and PI at 10 × 3 magnification. Left image: Annexin-stained (apoptotic) cells are depicted as green cells. PI-stained (dead) cells are depicted as red cells. Right image: example of the ImageJ function ‘find maxima’ results indicating apoptotic cells included in the results. Scale bar: 100 µm.

Next we investigated if disc flattening prior to injury had measurable effects on the amounts of apoptotic and dead cells 24 and 96 h after injury (Figure 5); representative images illustrating the method are given in Figure 5J. Having established the general effects of flattening in a systematic analysis including comparisons against flattening alone (above), subsequent experiments omitted the flattening alone group.

Across the full 250 µm disc thickness (Figures 5A–C), the flattening and injury groups displayed significantly increased numbers of Annexin-positive cells compared with controls at both 24 and 96 h after injury. Dead control cell numbers were significantly increased at 96 h. The apoptotic ratio was significantly elevated at 96 h in the flattening and injury group, compared with the 96 h control. Within the upper 90 µm disc region (Figures 5D–F), both the apoptotic cell amounts and apoptotic ratio were significantly higher in the flattening and injury groups, compared with controls at both 24 and 96 h after injury. Dead cell numbers did not differ significantly between groups. In the uppermost 44 µm disc region (Figures 5G–I), the flattening and injury groups again showed significantly increased numbers of Annexin-positive cells and significantly increased apoptotic ratios, compared with controls at both 24 and 96 h after injury. Dead cell numbers were significantly increased at 24 h after flattening and injury. Overall, these findings demonstrated that flattening prior to injury enhanced injury-induced apoptotic responses. The effects persisted up to 96 h post-injury, and the strongest effects were observed in the uppermost ∼44 µm of the tissue.

#### 3.5.3 Effect on proliferation

**FIGURE 6:**
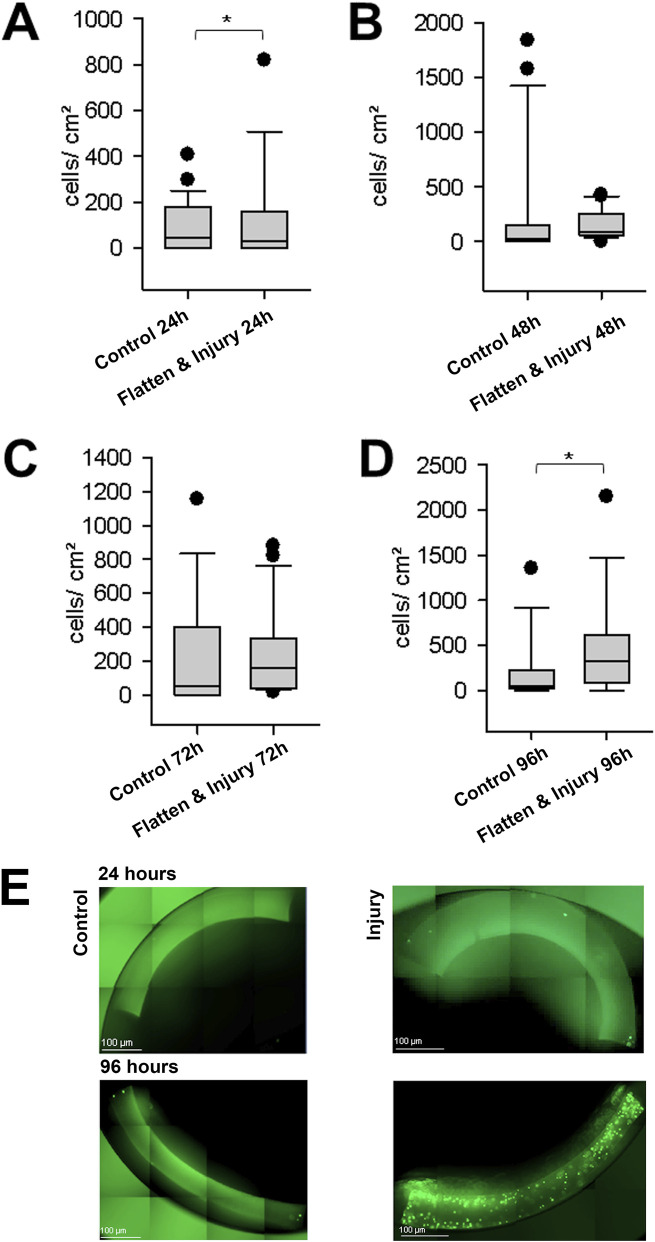
Proliferating cells after flattening of superficial zone discs followed by a single injurious compression (65%) 24, 48, 72, and 96 h after injury. Results for control discs and for flattened and injured discs at **(A)** 24 h **(B)** 48 h **(C)** 72 h, and **(D)** 96 h that were incubated in medium containing Click. It™-EdU, fixed and permeabilized on the respective days, showing proliferating cells as number of EdU-stained cells per disc per um^2^. Injury consisted of a single uniaxial unconfined compression (65% final strain, 100%/s strain rate). 24 h: control n = 24 (4 donors), injury n = 18 (4 donors); 48 h: control n = 21 (4 donors), injury n = 21 (4 donors); 72 h: control n = 17 (4 donors), injury n = 22 (4 donors); 96 h: control n = 18 (4 donors), injury n = 19 (4 donors). Here, *n* denotes the number of analyzed half-discs. The * indicates a significant difference between groups (p < 0.05) **(E)** Representative images of proliferation in superficial zone discs in control and injured discs after 24 and 96 h. Scale bar: 100 µm.

As a final step, this study investigated if flattening combined with injury had an effect on the number of proliferating cells in the full 250 µm disc thickness 24, 48, 72, and 96 h after injury (Figures 6A–D). Interestingly, the flattening and injury group showed significantly increased numbers of EdU-positive cells compared with controls at 24 (Figure 6A) and 96 h (Figure 6D), whereas no significant differences were observed at 48 or 72 h (Figures 6B,C). Representative images are shown in Figure 6E. Collectively, these findings demonstrated that flattening combined with injury significantly enhanced a temporally dynamic proliferative response within the full 250 µm superficial zone disc thickness, with the strongest effects observed at 96 h after injury.

## 4 Discussion

Long-standing challenges of *ex vivo* curling, swelling, and deformation of soft tissue explants compromise the application of mechanical loading models. Here, we introduce a solution to these challenges. ‘Mechanical flattening’, using a regression-guided flattening load computed from each disc’s swelling state, restores the thickness and uncurls each articular cartilage explant prior to injurious compression. Crucially, this enabled standardized injurious compression of the deformation-prone superficial zone, which many existing injury models simply remove, despite its clinical relevance as the most damage-susceptible and cell-dense zone. The flattening step significantly amplified injury effects such as reduced viability and increased apoptosis-associated signaling with damage concentrated in the top-most region of the superficial zone, likely through reduced strain heterogeneity. Indeed, swelling-induced curling introduces spatially uneven pre-strain and alters the explant’s surface contour, causing highly variable contact and heterogeneous stress fields during compression (Setton et al., 1998). Previous work has shown that the mechanical signature of injury - specifically the shape of the stress-vs-time curve - can accurately predict the extent of structural and biochemical damage in cartilage, particularly within the superficial zone (Rolauffs et al., 2013). This underscores that reproducible loading geometry is essential for generating interpretable and reproducible injury responses. By restoring flatness and native thickness prior to injury, our approach minimizes curvature-induced geometric variability and likely re-establishes a more uniform loading geometry with consistent boundary conditions across samples. This reduction in geometric heterogeneity should improve the reliability and predictive value of mechanical load or overload and may help concentrate strain within the most deformation-sensitive superficial tissues, thereby amplifying biologically meaningful injury responses. Notably, injury also induced S-phase entry indicating proliferation across the superficial zone, which few studies have reported so far, and whose detection was supported through the here introduced approach to mitigate *ex vivo* deformation. This simple, scalable deformation correction standardizes injurious compression, reveals biologically meaningful responses through amplifying injurious effects, and is transferable to other deformation-prone soft tissues.

At the core of this *ex vivo* deformation-mitigating approach is a nonlinear regression-derived equation that predicts the compressive load necessary to return swollen and curled tissue discs back to their original, pre-swelling thickness. The equation was established on 250 µm superficial zone cartilage discs through nonlinear regression analysis and yields the load required to flatten a disc to its original thickness while requiring T_2_ being the disc thickness at day 2 post-dissection. The purpose was not precise biomechanical prediction, but operational standardization of the flattening procedure across discs with varying degrees of swelling and curling. Despite the modest R^2^ (0.349) obtained in “Step 2: Predicting the flattening load (LT_0_) from swelling (T_2_ – T_0_)”, the model provides an empirically useful approximation of the load required to restore native geometry, rather than to fully explain the biomechanical variability associated with tissue swelling and deformation behavior. Importantly, the utility of this approach is supported by the overall experimental findings: flattening reproducibly restored disc geometry, flattening alone was largely non-injurious, and flattening prior to injury improved the consistency and detectability of superficial-zone injury responses. The transfer to other deformation-prone soft tissues could be achieved by measuring post-equilibration thickness and/or curling of a set of explants, computing the deviations from the native state, and fitting a regression model that maps these descriptors to the required flattening loads. In our case, because superficial-zone discs rapidly re-curled after load release, flattening and injury application were performed as a continuous workflow. While this was readily achievable within the present experimental setup, adaptation to other tissue types or loading systems may require additional optimization of workflow timing and mechanical handling. Thus, custom equations can be generated in a similar fashion that are specific to different laboratory practices, tissues, or scientific questions. However, transferability to other tissue types currently remains conceptual and will require experimental validation in additional deformation-prone tissues.

Previous studies, including work in our own laboratories (Rolauffs et al., 2010a; Imgenberg et al., 2013), have at times used a slow pre-compression step to ‘return’ explants to their nominal cutting thickness before injurious loading. This position-controlled approach differs fundamentally from the load-controlled, regression-guided flattening used here. Swelling-induced curling produces heterogeneous residual strains (Setton et al., 1998) and variable bending stiffness, such that two explants with identical cutting thickness may require markedly different forces to achieve true geometric flattening. A fixed piston position therefore does not guarantee restored flatness or uniform surface contact - particularly for thin superficial-zone explants - or when low-resolution calipers are used that compress the tissue and sample only a single point. Our calibration model explicitly links an explant’s swelling state to the load required to restore native geometry, providing a reproducible, quantifiable flattening step that avoids operator-dependent measurements and does not alter cell viability. To our knowledge, prior work has not evaluated or standardized the effects of pre-compression, nor demonstrated that flattening alone is non-injurious. Thus, the present load-prediction framework is not simply a reimplementation of earlier practices, but a calibrated and generalizable method for restoring geometry and improving the reproducibility of *ex vivo* injury loading.

Until now, post-injurious proliferation in healthy adult cartilage has received relatively little attention. Here we show that injurious compression alone, applied to mechanically mature adult articular cartilage using standardized uniaxial unconfined loading after correcting for swelling-induced deformation, leads to robust and spatially localized proliferation within the superficial zone up to 96 h after injury. Proliferating cells were detected using click-chemistry EdU incorporation, which marks DNA synthesis during S-phase. Only a few prior studies have examined cartilage proliferation after injurious compression, and most differ fundamentally from the present work. Increased proliferation has been shown after creating mechanical defects using invasive injury techniques, which differs from our technique of non-invasive injurious compression (Tew et al., 2000). Porter et al. applied unconfined injurious compression to juvenile bovine cartilage and used AmdU (5-(azidomethyl)-2′-deoxyuridine) click-chemistry labeling similar to the EdU (5-ethynyl-2′-deoxyuridine) labeling used in this study. However, they observed reduced proliferation (Porter et al., 2022), potentially due to age-related differences in baseline proliferative capacity and the greater injury susceptibility of juvenile cartilage (Kurz et al., 2004). Henson et al. reported chondrocyte proliferation in adult equine cartilage following impact injury by manually counting *de novo* cells in impacted cartilage treated with and without FGF-2, and in unimpacted cartilage treated with FGF-2. However, they did not compare impacted vs. unimpacted tissue in the absence of any growth factor (Henson et al., 2005). Wong et al. (2005) observed post-impact proliferation in adult rabbit cartilage using bromodeoxyuridine (BrdU) and flow cytometry but without zonal specificity and without comparisons to an unimpacted control. Additionally, studies of OA human adult cartilage have reported a low level of chondrocyte proliferation in OA cartilage (i.e., in late-stage disease) (Meachim and Collins, 1962; Rothwell and Bentley, 1973; Aigner et al., 2001) or distal to a focal OA lesion (Rolauffs et al., 2010b). Studies on early OA tissue usually describe chondrocyte clones. One of our studies attributed these chondrocyte clones to growth factors and specifically to FGF2, which we found to increase proliferation while inducing catabolic gene expression, which can potentially be linked to the onset of inflammation (Boehme and Rolauffs, 2018). Thus, the present study demonstrates injury-induced proliferation in healthy adult cartilage without exogenous growth factors, and with clear localization to the superficial zone, revealing a proliferative response that has been largely overlooked due to methodological and biological differences in prior models, warranting further mechanistic investigation.

However, the observed increase in EdU-positive cells following flattening and injury should be interpreted cautiously, as proliferative responses in adult articular cartilage are typically limited and mechanistically incompletely understood. Accordingly, EdU incorporation in the present study was interpreted as S-phase entry consistent with proliferative activation rather than definitive evidence of completed proliferation or regenerative tissue formation. Importantly, the enhanced detection of EdU-positive cells may partly reflect improved geometric standardization and more reproducible injury application following flattening, which likely reduced variability in superficial-zone loading conditions and thereby increased the detectability of injury-associated cellular responses. In this regard, improved detection of biologically relevant responses through reduction of geometrically induced experimental variability represents an additional conceptual advantage of the presented flattening approach. Notably, the observed EdU-positive response occurred in adult, mechanically mature cartilage without exogenous growth factors and was spatially associated with the injury-exposed superficial zone, supporting the biological relevance of the finding. Taken together, the combined observation of early apoptosis and later S-phase entry reveals a biphasic superficial-zone response to mechanical overload. This coordinated apoptotic–proliferative pattern is rarely captured in adult cartilage, in part because curvature-induced load heterogeneity in prior *ex vivo* systems can obscure such spatially resolved responses. By correcting deformation and thereby improving loading fidelity, the present model helped expose a temporal sequence of mechanobiologically induced events - early cell loss followed by localized proliferative activity - that may underlie early phase PTOA pathogenesis.

To validate the approach of pre-injuriously flattening curled and swollen discs, we conducted a proof-of-concept study, first demonstrating that the pre-injury flattening of curled discs by itself had no effect on cell viability, compared to controls and injured discs. Equally important, applying flattening prior to injurious compression to account for post-equilibrium tissue swelling and curling significantly enhanced the injury model’s performance, especially in delivering consistent injury responses. Flattened, injured discs exhibited significantly greater cell death and reduced viability than non-flattened controls (p < 0.001), particularly within the uppermost 44 µm of the superficial zone, underscoring the method’s ability to produce biologically relevant injury responses. These results align with prior findings that injury-induced cell death is concentrated in the superficial zone and increases with higher mechanical strains (Ewers et al., 2001; Rolauffs et al., 2010a; Chen et al., 2003; Lewis et al., 2003). Importantly, this study also revealed a sustained increase in apoptosis-associated signaling throughout the entire 250 µm disc thickness for up to 96 h. However, Annexin V staining was assessed in mechanically injured *ex vivo* cartilage tissue and apoptosis-associated membrane changes should be interpreted cautiously, as transient mechanically induced membrane perturbation cannot be fully excluded. Whereas other studies did not focus solely on the superficial zone, this is consistent with prior *ex vivo* articular cartilage studies using similar injurious compressive strains. These studies conducted in newborn (Patwari et al., 2004; Frank et al., 2000), juvenile, and adult canine and bovine cartilage (Chen et al., 2001), as well as mature bovine osteochondral explants (Rosenzweig et al., 2012; Clements et al., 2001; Morel and Quinn, 2004) likewise observed increased chondrocyte apoptosis following injurious compression. Although we did not compare 50% and 65% strains in non-flattened discs, the fact that clear depth-dependent differences emerged at 24 h under flattened conditions suggests that geometric correction does not obscure—but may help reveal—strain-dependent injury responses in the superficial zone. Together, our findings and the discussed points emphasize the robustness and relevance of the here introduced approach as an improved *ex vivo* superficial zone model for PTOA research. The fact that the superficial zone contains the majority of cells (Crockett et al., 2007), is the first region affected by shear or blunt trauma (Ewers et al., 2001; Rolauffs et al., 2010a; Jones et al., 2009; Bartell et al., 2015), and is highly susceptible to early degeneration (Bauer and Jackson, 1988; Rolauffs et al., 2010a; Guo et al., 2025), yet is often excluded in previous injurious compression studies (Patwari et al., 2003; Patwari et al., 2004; Sui et al., 2009; Behrendt et al., 2016; Stevens et al., 2009; Kurz et al., 2001), only emphasizes the importance of this improved *ex vivo* superficial zone model for PTOA research since it preserves the clinically important superficial zone. Moreover, our findings validate the model’s utility for studying early PTOA-related post-injury changes including viability loss, apoptosis-associated signaling, and superficial zone-specific proliferation. This model establishes a foundation for future superficial zone explant injurious compression studies for understanding superficial zone responses to injury, exploring PTOA progression, and testing novel interventions that target the early events in the upper regions of the superficial zone.

Beyond articular cartilage, this method, centered around a nonlinear regression-derived equation that predicts the load needed to restore swollen, curled tissue to its original thickness, offers a versatile, reproducible approach for investigating mechanical load responses that can easily be applied to other deformation-prone soft tissues and enhances our understanding of cell and tissue responses to load and subsequent events that ultimately contribute to clinical disease.

## Data Availability

The raw data supporting the conclusions of this article will be made available by the authors, without undue reservation.
